# Protein‐Ratio Rheostats: Integrating Stoichiometry, Abundance, and Binding Affinity in Cellular Function

**DOI:** 10.1002/bies.70195

**Published:** 2026-10-08

**Authors:** Subbaya Subramanian, Reena V. Kartha

**Affiliations:** ^1^ Department of Surgery University of Minnesota Medical School Minneapolis Minnesota USA; ^2^ Masonic Cancer Center University of Minnesota Minneapolis Minnesota USA; ^3^ Center For Immunology University of Minnesota Minneapolis Minnesota USA; ^4^ Department of Experimental & Clinical Pharmacology College of Pharmacy University of Minnesota Minneapolis Minnesota USA

**Keywords:** binding affinity, molecular assembly, protein stoichiometry, protein–protein interactions, protein‐ratio rheostat, relative protein abundance

## Abstract

Cellular function is frequently interpreted in terms of gene presence, protein abundance, and dose–response relationships. Yet interacting proteins operate within binding and assembly regimes in which relative abundance can alter which molecular complexes predominate. We propose a protein‐ratio rheostat framework in which functional output depends jointly on component ratios, absolute concentrations relative to binding affinities, interaction stoichiometry, and in multivalent systems, valency and cooperativity. The same protein ratio can therefore generate different molecular and functional states under different concentration or affinity regimes. We develop testable predictions of this framework, illustrate its application to competitive protein interactions and biomolecular condensates, and outline experimental strategies for identifying composition‐sensitive behavior within defined abundance and affinity regimes.

## Graphical abstract

Relative protein composition can act as a rheostat by altering molecular assembly within defined abundance and binding regimes. MicroRNAs, translation, and selective degradation can reshape protein composition, influencing signaling, apoptosis, development, and disease. Keywords: protein ratio, stoichiometry, binding affinity, molecular assembly, microRNA.

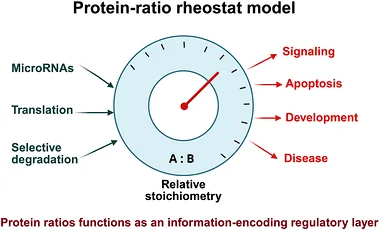

## 1 Introduction

When a painter seeks a particular shade of green, she does not simply reach for “green” paint; she mixes yellow and blue in a deliberate ratio. A 10:90 mixture produces a different shade from a 50:50 mixture, although both contain the same pigments. Cellular systems can operate similarly: functional outcomes may depend not only on which proteins are present or how abundant they are, but also on their relative abundance within interacting molecular assemblies.

Classical molecular genetics established the importance of gene regulation and gene products in determining phenotype [1]. Quantitative cell biology subsequently extended this view by considering molecular abundance, variability, and absolute copy number [2, 3]. For interacting molecules, however, relative abundance cannot be interpreted independently of absolute concentration and binding affinity. Whether a change in component ratio alters complex formation depends on component concentrations relative to the dissociation constant, *K*_D_. The same ratio can therefore produce different occupancies across concentration regimes; as component concentrations increasingly exceed *K*_D_, assembly becomes more constrained by the limiting component. The biological significance of a protein ratio is therefore regime dependent [4, 5].

This regime dependence is evident in competing endogenous RNA networks, where appreciable cross‐regulation occurs only within particular ranges of transcript and microRNA abundance [6, 7, 8, 9, 10, 11]. These observations illustrate a broader principle: relative abundance becomes mechanistically informative only in the context of the concentrations, affinities, and interaction architecture that determine which complexes or assemblies form.

Stoichiometry is a key determinant of molecular interactions and complex assembly [12, 13, 14]. The protein‐ratio rheostat framework therefore does not replace biochemical or systems‐level models but makes relative composition an explicit experimental coordinate linking molecular abundance to assembly and function. Relative protein abundance may provide additional functional information when interpreted together with absolute concentration and interaction energetics. A protein ratio is neither a substitute for concentration nor an independent determinant of molecular state; its significance lies in whether it shifts the complexes or assemblies that predominate within a defined biochemical regime. This framework can therefore be tested across transcription, signaling, apoptosis, receptor assembly, and disease.

Cells can establish and remodel relative protein abundance through partial repression by microRNAs [15, 16, 17, 18], translational control and selective protein degradation [19, 20, 21, 22, 23, 24], and alternative isoform production or regulated intracellular trafficking [25, 26, 27]. These mechanisms can change the composition of molecular pools available for interaction without eliminating individual components. We therefore treat protein ratio as an experimentally measurable coordinate of composition whose functional significance emerges from the concentration, affinity, stoichiometry, and assembly regime in which the interacting components operate.

## 2 Conceptual Framework: From Abundance to Composition‐Dependent Function

Classical genetic experiments establish causality by testing whether loss or gain of a gene product alters phenotype [1]. These approaches remain fundamental, but quantitative changes in expression or dosage can also produce graded or context‐dependent effects [2].

Quantitative approaches extend this view by considering molecular abundance, kinetics, and dose–response relationships. Classical mass‐action and fractional‐occupancy models provide a quantitative basis for this interpretation, because molecular occupancy varies with concentration relative to the dissociation constant [28]. These relationships are often nonlinear, and proteins may also participate in heterodimers, multisubunit complexes, competitive interactions, or higher‐order assemblies [4, 5, 12, 13, 14]. Changing the abundance of one component can therefore alter not only its own availability but also the distribution of its partners among alternative molecular states.

The protein‐ratio rheostat framework adds relative composition to this quantitative description. As the abundance of one component changes relative to another, the distribution of complexes or assemblies can shift even when all components remain present. The resulting relationship between component ratio and biological output has no universal form: depending on binding affinity, stoichiometry, competition, cooperativity, feedback, and total abundance, responses may be gradual, saturating, sigmoidal, threshold‐like, or non‐monotonic. Relative abundance is therefore not the mechanism itself; its functional significance arises from the interaction architecture that converts compositional changes into altered occupancy, complex distribution, or higher‐order assembly [12, 29, 30, 31, 32, 33, 34].

The paint analogy captures composition only. In biological systems, binding, competition, cooperativity, and collective assembly can transform progressive changes in composition into nonlinear or abrupt functional responses. Relative abundance should therefore be treated as one measurable dimension of regulation, interpreted together with absolute concentration and the interactions that determine molecular assembly. Figure 1 schematically summarizes these dimensions and illustrative response topologies.

**FIGURE 1:**
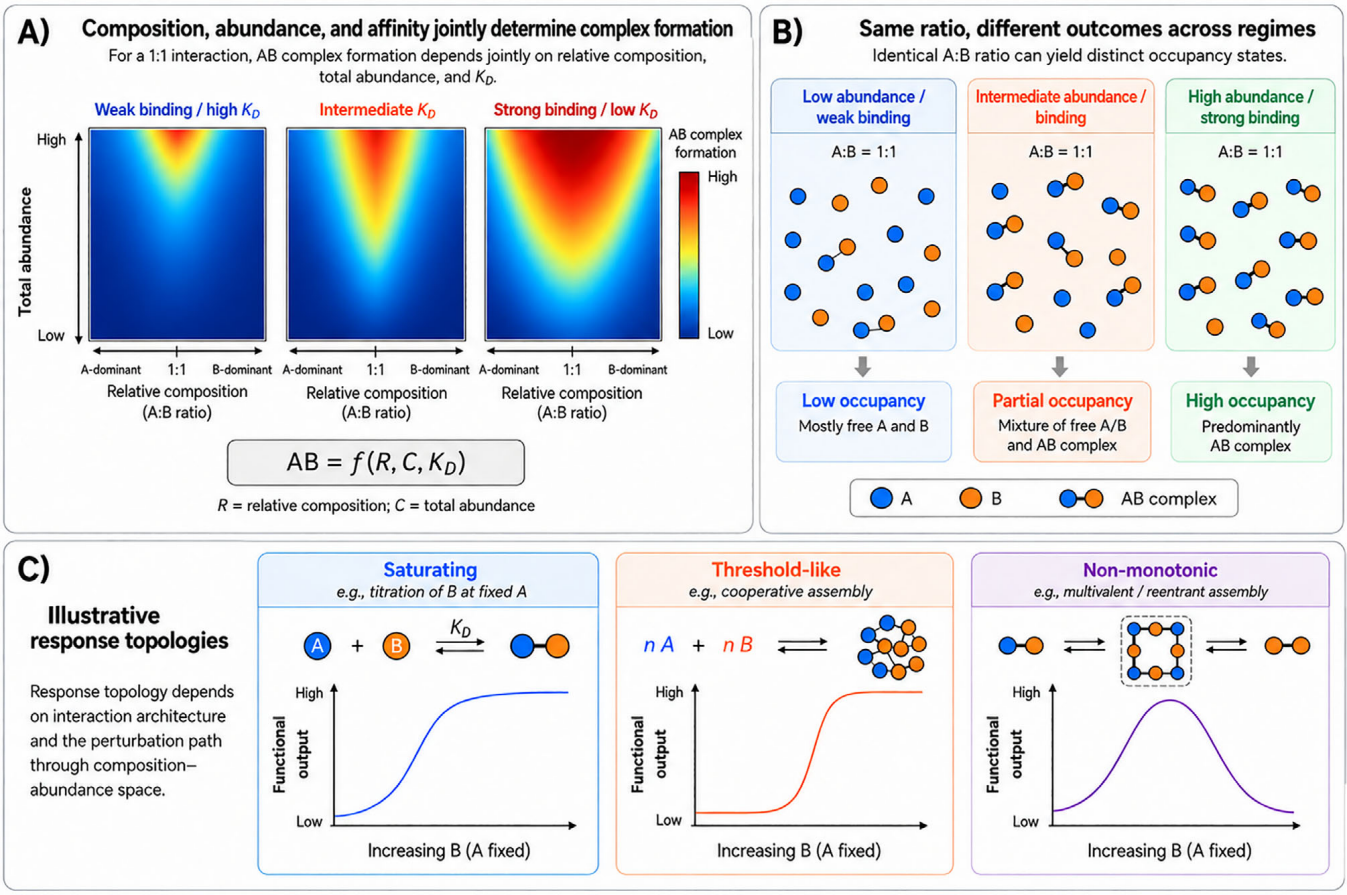
Conceptual representation of the protein‐ratio rheostat framework. (A) For a simple 1:1 interaction, AB complex formation depends jointly on relative composition, total component abundance, and binding affinity. Illustrative response maps for weak binding/high *K*_D_, intermediate *K*_D_, and strong binding/low *K*_D_ show that complex formation is favored near balanced *A:B* composition but varies substantially with total abundance and binding strength. Ratio (*R*) and total abundance (*C*) therefore provide coordinates of the underlying component‐concentration space, such that AB  =  *f*(R, C,K_*D*_). (B) The same *A:B* ratio can correspond to different molecular occupancy states under different biochemical regimes. At a fixed *A:B* ratio of 1:1, low abundance and weak binding yield predominantly free A and B, an intermediate regime yields partial occupancy with both free components and AB complex, and high abundance and strong binding yield predominantly AB complex. Thus, ratio alone does not determine molecular assembly state. (C) Different interaction architectures can generate distinct response topologies under the same illustrative perturbation path. When B is increased while A is held fixed, a simple pairwise interaction may produce a saturating response, cooperative assembly may generate threshold‐like behavior, and multivalent or reentrant assembly may produce a non‐monotonic response. These curves are schematic rather than universal quantitative relationships; the observed topology will depend on interaction architecture, abundance, binding parameters, cooperativity, and valency. This figure was generated using the institutionally approved OpenAI tool.

## 3 Quantitative Basis of the Protein‐Ratio Rheostat

For a simple 1:1 interaction in which proteins *A* and *B* form a complex (*A*+*B*⇌*AB*), equilibrium binding is described by *K*_D_ *=* [*A*][*B*]/[*AB*], where *K*_D_ is the dissociation constant. In terms of the total concentrations *A*_T_ and *B*_T_, the equilibrium concentration of the complex is:

$$\left[AB\right]=\frac{A_{T}+B_{T}+K_{D}-\sqrt{{\left(A_{T}+B_{T}+K_{D}\right)}^{\wedge }2-4A_{T}B_{T}}}{2}$$

Thus, complex formation depends on absolute concentrations as well as their relative abundance. Accordingly, the *A*:*B* ratio alone does not determine how much *AB* complex forms. Two systems with the same *A:B* ratio can have markedly different complex occupancies when their absolute concentrations differ relative to *K*_D_. At concentrations low relative to *K*_D_, occupancy remains strongly concentration‐dependent; as concentrations increasingly exceed *K*_D_, binding becomes more stoichiometrically constrained, and the limiting component becomes more influential. The functional significance of a protein ratio therefore depends on the concentration‐affinity regime in which the interaction occurs [4, 5, 35].

We define a protein‐ratio rheostat as an interacting molecular system in which changes in relative component abundance alter the distribution of functional complexes or assemblies within a defined concentration‐affinity regime. The biologically relevant variable is therefore not the numerical ratio in isolation, but the molecular state that composition generates under the conditions governing interaction and assembly.

Importantly, in a two‐component system, ratio and total abundance reparameterize the two component concentrations rather than serving as independent molecular variables. The utility of the ratio coordinate is therefore mechanistic: it makes composition‐sensitive interactions explicit and tests whether interaction or assembly terms capture behavior better than models that treat component abundances independently.

Different interaction architectures can generate distinct ratio–response relationships. In simple pairwise systems, changing component ratio may progressively alter the fraction of molecules in complex. Competition for a shared partner can amplify or diminish the effect of relative abundance according to binding affinity, while cooperative or multimeric interactions can generate sigmoidal or threshold‐like responses. Multivalent assemblies can instead produce non‐monotonic behavior in which assembly is maximal over an intermediate range of component ratios. These patterns are not intrinsic properties of the ratio; they emerge from the interaction architecture that translates relative abundance into occupancy, complex composition, or higher‐order assembly [12, 29, 30, 31, 32, 33, 34].

The protein‐ratio rheostat framework therefore complements rather than replaces concentration‐based models. Absolute abundance establishes the concentration regime, binding affinity influences which interactions are favored, and relative abundance constrains the availability of competing or cooperating components. Their contributions can be examined experimentally by varying interacting components across a concentration matrix, altering interaction affinity where feasible, and measuring the resulting changes in complex formation and functional output. Relative composition and total abundance can then be treated as derived coordinates of the experimentally sampled concentration space.

## 4 How the Ratio‐Rheostat Framework Differs From Existing Models

The ratio‐rheostat framework builds on established concepts in biochemical thermodynamics, stoichiometric complex assembly, systems biology, and dose–response modeling rather than replacing them [4, 5, 12, 13, 35, 36, 37, 38]. Its contribution is to make relative composition an explicit experimental coordinate linking molecular abundance to complex formation and downstream function. Classical binding models describe how concentration and affinity determine occupancy, stoichiometric models define constraints on complex assembly, and network models relate molecular interactions to system‐level outputs. The ratio‐rheostat framework connects these levels by testing whether changes in the relative availability of interacting components redistribute functional complexes or assemblies within a defined biochemical regime. Quantitative studies of steroid receptor‐DNA interactions provide a concrete precedent for linking interaction energetics to functional transcriptional output using explicit binding models [35].

This distinction is particularly important when family members, isoforms, or subunits compete for a shared binding partner. Equal abundance of two competitors need not produce equal occupancy because their binding affinities may differ. A less abundant, higher‐affinity component can therefore occupy more of the shared partner than a more abundant, weaker‐binding competitor, whereas changes in relative abundance may have little effect when one interaction is strongly favored or the shared partner is limiting. Functional competition in such systems therefore reflects the combined effects of relative abundance, binding affinity, and absolute concentration [4, 5]. The framework therefore shifts interpretation from measured protein ratios to the molecular states associated with those measurements. A change in relative abundance is mechanistically informative when it redistributes interacting components among functionally distinct complexes or assemblies. Its value can therefore be tested by asking whether models that explicitly represent relative composition, interaction terms, or measured complex occupancy explain or predict system behavior better than models that treat component abundances independently.

## 5 Biological Examples in Which Relative Composition Shapes Function

The biological relevance of the ratio‐rheostat framework is most apparent in systems where changes in the relative availability of interacting components redistribute molecular complexes or assemblies. The examples below illustrate distinct interaction architectures rather than a universal ratio–response relationship.

Transcription factor dimers provide a direct example. Families such as JUN/FOS (AP‐1) and MYC/MAX depend on dimerization for DNA binding and transcriptional activity [39, 40, 41]. The distribution of transcriptionally active dimers reflects the relative availability of alternative partners together with their dimerization and DNA‐binding affinities. Changing one family member can therefore redistribute complexes, so transcriptional output is not predictable from the JUN or MYC ratio alone. The NFκB family illustrates the same principle in a more regulated setting: dimers including p50/RelA, p50/p50, and RelB/p52 have distinct transcriptional properties [42], and their distribution also depends on inhibitor interactions, localization, activation state, and turnover. Relative abundance therefore influences function by contributing to the distribution of alternative transcription‐factor complexes.

The BCL2 family provides a mechanistically informative example because mitochondrial apoptosis is controlled by interactions among antiapoptotic proteins, BH3‐only proteins, and the effectors BAX and BAK. Antiapoptotic proteins can sequester specific BH3‐only activators or activated BAX and BAK, while BH3‐only proteins differ in their binding preferences for individual antiapoptotic partners. Consequently, the same aggregate pro‐ to antiapoptotic ratio can produce different responses when component identities, concentrations, or affinities differ. The relevant molecular state is therefore the distribution of specific inhibitory and activating complexes generated by relative abundance and partner‐specific interactions [43, 44, 45].

Ligand‐gated ion channels illustrate composition‐dependent assembly of multisubunit complexes. GABAA and nicotinic acetylcholine receptors are assembled from multiple subunit types, and alternative compositions or stoichiometries can produce receptors with distinct conductance, ligand sensitivity, pharmacology, and localization [46, 47, 48, 49, 50]. Relative subunit availability influences the assemblies that are accessible, while intrinsic assembly preferences and cellular constraints determine which complexes form. These systems distinguish the abundance ratio of available subunits from the stoichiometry of the functional receptor assembled from them.

Biomolecular condensates extend the framework beyond pairwise or fixed‐stoichiometry complexes. Landmark studies established that membrane‐less cellular compartments can behave as liquid‐like phases and can arise through liquid–liquid phase separation, providing a physical basis for composition‐dependent assembly in cells [29, 34, 51, 52]. Because they assemble through multivalent interactions, their behavior can depend on component concentration, relative stoichiometry, valency, polymer length, charge, and collective interaction strength. In protein‐RNA systems, increasing RNA relative to protein can initially promote intermolecular connectivity and phase separation, whereas further increases can reduce productive cross‐linking and suppress condensation. Assembly can therefore be maximal over an intermediate composition range, producing non‐monotonic or reentrant behavior [30, 32, 33]. Related studies of phase‐separated signaling clusters further show that component stoichiometry can alter biochemical activity within condensates [31].

Condensates also define an important boundary of the framework: unlike simple pairwise interactions, they generally cannot be described by a single *K*_D_ but require consideration of phase boundaries and collective interaction parameters. Relative composition is therefore only one coordinate within a higher‐dimensional assembly landscape [12, 29, 30, 31, 32, 33, 34].

Together, these examples illustrate distinct routes by which relative abundance can influence function: redistribution among alternative dimers, partner‐specific sequestration and activation, multisubunit receptor assembly, and multivalent phase separation. In each case, the mechanistically relevant question is whether changing relative composition redistributes functional complexes or assemblies within the relevant concentration, affinity, and interaction regime.

## 6 Mechanisms That Establish and Reshape Protein Ratios

Cells can alter the relative abundance of interacting proteins through multiple regulatory mechanisms. MicroRNAs are one example because they frequently produce partial rather than complete target repression, allowing interacting components to be rebalanced without eliminating them. Such changes can shift a system into a different concentration, composition, or binding regime.

Because individual miRNAs can regulate multiple mRNAs and individual mRNAs can be regulated by several miRNAs [15, 16, 17, 18, 53], miRNA‐mediated repression can coordinate abundance changes across molecular networks. When regulated targets participate in the same pathway or complex, these changes can alter relative and absolute abundance. Their functional significance depends on whether the resulting compositional shift redistributes the relevant complexes or assemblies.

miRNA expression and processing can respond to developmental, stress, and environmental signals, allowing protein composition to change dynamically [54, 55]. For example, miR‐15 and miR‐16 regulate BCL‐2 expression and can thereby alter apoptotic susceptibility [56]. Such posttranscriptional regulation can shift the balance among interacting proteins without eliminating individual components.

Relative abundance can also be reshaped indirectly through competition for shared miRNAs. When competing endogenous RNAs are sufficiently abundant relative to the available miRNA pool, changes in one transcript can alter miRNA availability and thereby affect repression of other targets [6, 7, 8, 9, 10, 11, 57, 58]. The magnitude of this effect depends on transcript and miRNA abundance, binding‐site affinity, and regulatory context.

Other mechanisms can reshape relative protein abundance independently of miRNAs. Selective protein degradation alters component lifetime and abundance [20, 21, 22, 23, 24], while alternative splicing changes isoform production with distinct or competing functions [25, 59]. Translational regulation, RNA‐binding proteins, differential stability, and intracellular trafficking can further modify the molecular pools available for interaction [19, 20, 26, 27]. Together, these processes allow relative composition to vary across time, cellular compartments, and physiological states.

The relevance of these regulatory mechanisms lies not simply in changing expression, but in altering the composition of interacting molecular pools. A regulatory perturbation becomes mechanistically informative when the resulting change in relative abundance redistributes functional complexes or assemblies and changes downstream output. The framework thereby links mechanisms that establish protein abundance to the interactions that translate composition into function.

## 7 Predictions and Testable Consequences of the Protein‐Ratio Rheostat Framework

The quantitative framework generates experimentally testable predictions that distinguish effects of relative composition from those of absolute abundance and binding affinity. The central prediction is not that protein ratio uniquely determines biological output, but that within defined interaction regimes, explicitly considering relative composition can reveal composition‐sensitive behavior arising from the interactions that connect component concentrations to molecular assembly and functional output.

### 7.1 Prediction 1: The Same Component Ratio Can Produce Different Molecular States at Different Absolute Concentrations

For a simple *A+B⇌AB* interaction, maintaining the same *A:B* ratio while varying the absolute concentrations of both components should alter complex occupancy outside a fully stoichiometrically constrained regime. Thus, a 1:1 *A:B* ratio at concentrations well below *K*_D_ need not produce the same occupancy or functional output as the same ratio at concentrations near or above *K*_D_. This directly tests whether ratio alone is sufficient to describe the system [4, 5].

### 7.2 Prediction 2: Composition‐Sensitive Behavior Should Emerge Across Coordinated Perturbations of Interacting Components

Rather than relying on a single constant‐total‐abundance trajectory, the concentrations of interacting components should be varied across a matrix of conditions. If molecular assembly or functional output depends on how component abundances are combined, the resulting response surface will reveal composition‐sensitive regions that are not apparent from varying either component alone. Relative composition and total abundance can then be used as derived coordinates to describe this experimentally sampled concentration space.

### 7.3 Prediction 3: Changing Binding Affinity Should Alter the Functional Consequence of a Fixed Protein Ratio

Mutations, posttranslational modifications, or other perturbations that alter interaction affinity can redistribute complexes without changing component abundance. In competitive systems, the same measured ratio can therefore correspond to different molecular states when binding affinities differ. This prediction tests whether the functional consequence of relative abundance depends on interaction energetics rather than abundance alone [4, 5].

### 7.4 Prediction 4: Changes in Molecular Assembly Should Mediate the Relationship Between Relative Abundance and Phenotype

If relative abundance influences function through complex formation or redistribution, changes in complex occupancy or composition should track functional output more closely than the abundance of either component alone. Perturbations that restore the relevant assembly state should also restore the associated functional output, at least in part, even if the original component concentrations are not fully reestablished. Demonstrating such an intermediate assembly state would provide mechanistic evidence that relative abundance operates through molecular interaction rather than association alone.

### 7.5 Prediction 5: Multivalent Systems Can Generate Non‐Monotonic Composition–Response Relationships

In condensates and other higher‐order assemblies, increasing one component may initially promote intermolecular connectivity but inhibit assembly once binding sites become saturated or productive cross‐linking is reduced. Functional or structural output may therefore be maximal over an intermediate composition range and decline when one component becomes excessive. The biologically relevant assembly state may thus occupy only a restricted region of composition space [12, 29, 30, 31, 32, 33, 34].

These predictions also define conditions under which the protein‐ratio rheostat framework would add little explanatory value. If system behavior is adequately explained by individual component concentrations and interaction affinity, if coordinated perturbations across the concentration space do not reveal composition‐sensitive changes in molecular assembly or functional output, or if measured assembly states do not track the proposed interaction‐dependent mechanism, then a ratio‐rheostat interpretation is unnecessary. The framework is therefore testable rather than universal.

## 8 Experimental Approaches to Demonstrate and Measure Ratio Rheostats

Demonstrating a protein‐ratio rheostat requires linking absolute and relative component abundance to molecular complex occupancy or composition and downstream functional output. Whenever feasible, interaction affinity should also be measured or estimated under biologically relevant conditions. Quantitative proteomics, targeted mass spectrometry, or single‐cell proteomic approaches can measure abundance [60, 61, 62, 63, 64], whereas FRET or BRET, bimolecular fluorescence complementation (BiFC), quantitative co‐immunoprecipitation, proximity‐labeling methods, and native mass spectrometry can assess interactions or assembly states [65, 66, 67, 68]. No single method is appropriate for every system; the critical requirement is to connect measured relative abundance to the molecular assembly that mediates function.

The critical experimental design is to sample the joint concentration space of the interacting components rather than infer composition dependence from single‐component perturbations. In a two‐component system, A and B can be varied across a matrix spanning low, intermediate, and high abundance. The resulting response surface can be analyzed in terms of the individual component concentrations and, where useful, reexpressed using relative composition and total abundance as derived coordinates. This approach identifies regions in which molecular assembly or functional output depends on the combination of component levels without assuming that equal total protein abundance imposes an equivalent cellular burden.

Binding affinity provides a complementary experimental dimension. Interaction‐interface mutations, posttranslational modifications, or other perturbations can alter affinity while component abundance is monitored. If the same *A:B* ratio produces different complex occupancies or functional outputs after affinity is changed, the effect of relative composition is interaction‐regime dependent rather than a property of the ratio alone. In competitive systems, measurements should determine how alternative partners redistribute among shared binding sites as abundance and affinity are varied.

Controlled perturbations can generate the required experimental range. Tunable promoters, inducible expression systems, CRISPRi/a [69], degron or dTAG approaches [70], and selected posttranscriptional perturbations can vary interacting components individually or in combination. Combinatorial perturbations should span multiple levels of each component while absolute abundance is monitored. Constant‐total‐abundance or constant‐ratio trajectories may provide informative slices through this response surface where technically feasible, but neither is required nor sufficient on its own to establish composition‐sensitive behavior. By contrast, single‐component overexpression or depletion changes multiple coordinates simultaneously and therefore cannot by itself establish composition dependence.

Functional readouts should reflect the molecular system and, whenever possible, be measured together with complex formation. Phosphoproteomic profiles can quantify downstream pathway activity [71], while apoptosis, receptor signaling, electrophysiological responses, transcriptional output, or other system‐specific phenotypes can provide functional endpoints. Spatial measurements may also be necessary because whole‐cell abundance may not reflect the local concentrations available for interaction [63]. The strongest evidence for a protein‐ratio rheostat would establish a linked sequence in which controlled changes in relative composition alter molecular assembly, and the resulting assembly state predicts downstream phenotype.

Computational modeling can test whether explicitly representing composition‐sensitive interactions improves explanatory or predictive performance. Mechanistic models can incorporate component concentrations, binding affinity, competition, cooperativity, and other experimentally supported parameters, while empirical response surfaces can represent ratio and total abundance as convenient coordinates of the same underlying concentration space. Models that treat component abundances independently should be compared with models that include interaction terms or measured complex occupancy and evaluated against independent perturbation data. This model‐comparison strategy makes the protein‐ratio rheostat framework quantitatively testable rather than a descriptive interpretation of correlated abundance changes [36, 37, 38].

## 9 Implications for Experimental Interpretation and Intervention

The protein‐ratio rheostat framework has implications for interpreting molecular perturbations in basic biology. Systems composed of family members, isoforms, or multisubunit complexes are defined not only by individual component abundance but also by relative availability and the interactions that determine which complexes or assemblies form. Deletion, depletion, or overexpression of one component can therefore alter phenotype both directly and by redistributing shared partners or alternative assemblies. Such perturbations should be interpreted in the context of the interacting system rather than attributed solely to the manipulated protein.

The same principle may apply to disease states in which function depends on competition among isoforms, paralogs, or opposing regulatory proteins. In cancer, changes in the relative abundance of functionally distinct isoforms can favor states associated with proliferation, survival, or other malignant phenotypes [45, 59, 72]. In selected systems, relative‐abundance measurements may therefore provide a compact measure of molecular composition that is not apparent from interpreting either component in isolation. However, empirical evidence must show whether such measurements improve diagnostic or prognostic performance.

Therapeutic implications follow from the same mechanistic constraint. If a pathological phenotype results from redistribution of interacting components among functionally distinct complexes, interventions that restore the relevant assembly state may warrant investigation. Such strategies could alter isoform abundance, protein stability, or upstream regulators of interacting components. The objective would not be to restore a numerical ratio itself, but to restore the molecular state associated with normal function. Evidence for therapeutic rebalancing would therefore require linking the intervention to altered complex composition or occupancy and improved phenotype. Clinical development of PROTAC degraders provides a concrete example that targeted modulation of protein stability can be therapeutically feasible; in the phase III VERITAC‐2 trial, the estrogen receptor degrader vepdegestrant prolonged progression‐free survival compared with fulvestrant in patients with ESR1‐mutated, ER‐positive/HER2‐negative advanced breast cancer [73].

Synthetic biology provides a complementary setting for prospective testing. Engineered systems can independently control the relative expression of interacting components and other experimental variables, allowing direct measurement of how composition influences assembly and output [74, 75]. Such systems also provide tractable models for systematically sampling component concentrations and testing how composition influences molecular assembly or functional output.

The broader implication is not that cellular regulation should be redefined around ratios, but that relative composition should be considered explicitly when function depends on competing, cooperating, or multivalent interactions. Incorporating composition alongside absolute abundance, binding affinity, and assembly state may improve interpretation of perturbation experiments and identify systems in which altered molecular composition contributes to disease or provides an experimental or therapeutic opportunity.

## 10 Challenges, Caveats, and Limitations

The pigment‐mixing analogy illustrates compositional variation only. Biological components differ fundamentally because they interact with finite affinities, compete for partners, undergo cooperative or allosteric transitions, occupy distinct cellular compartments, and may operate outside thermodynamic equilibrium. A protein ratio therefore has no universal functional meaning outside the biochemical and cellular regime in which it is measured.

A principal limitation of ratio‐based interpretation is that measured abundance is not equivalent to molecular occupancy. The same *A:B* ratio can generate different amounts of AB complex when absolute concentrations differ relative to *K*_D_, while similarly abundant proteins can contribute unequally to complex formation when their affinities for a shared partner differ. Whole‐cell protein measurements may also poorly approximate the free concentrations available for interaction because components can be sequestered, compartmentalized, incorporated into alternative complexes, or posttranslationally modified. A measured abundance ratio should therefore not be assumed to represent the ratio of molecules available to interact [4, 5].

Equilibrium binding affinity may itself be insufficient to predict function in systems dominated by kinetics or active regulation. Association and dissociation rates, protein turnover, trafficking, posttranslational modification, feedback, and energy‐dependent processes can alter occupancy even when component abundance and equilibrium affinity are unchanged. The ratio‐rheostat framework is therefore most directly applicable when the interactions linking relative abundance to assembly can be identified and measured. In strongly nonequilibrium systems, kinetic models may be more informative than equilibrium descriptions based on *K*_D_ [4, 5, 37].

Measurement presents an additional challenge because relative abundance can vary across cells, subcellular compartments, and time, whereas many proteomic measurements report population‐averaged or whole‐cell abundance. Such measurements may obscure the local molecular environment in which an interaction occurs, particularly at low copy number, where stochastic fluctuations increase cell‐to‐cell variation, or during transient signaling responses [37]. Single‐cell, spatial, or time‐resolved measurements may therefore be required to determine whether the measured composition reflects the molecular pool that generates the phenotype [60, 61, 62, 63].

The framework is also expected to add little information when one component is present in sufficient excess that further changes in its abundance do not appreciably alter the distribution of functional complexes, or when another variable primarily determines output. More generally, a ratio‐based interpretation is unnecessary when absolute abundance, binding affinity, or another established mechanism adequately explains the observed behavior. An association between protein ratio and phenotype is therefore insufficient; the relevant test is whether explicitly accounting for composition‐sensitive interactions or assembly improves mechanistic explanation or prediction compared with treating the measured components independently.

Multivalent assemblies present a distinct limitation to pairwise descriptions. Biomolecular condensates generally cannot be represented by a single *K*_D_; their formation and composition depend on variables including total concentration, relative stoichiometry, valency, polymer architecture, charge patterning, and collective interaction strength. Such systems require phase diagrams or multivalent interaction models rather than simple pairwise binding descriptions [12, 29, 30, 31, 32, 33, 34] Relative composition remains relevant, but only within the higher‐dimensional assembly landscape defined by these parameters.

These limitations establish the intended boundaries of the protein‐ratio rheostat framework. Relative abundance should be treated as a mechanistic variable only when changes in composition can be linked to altered molecular occupancy, complex distribution, or higher‐order assembly. The framework is therefore not an alternative to concentration‐, affinity‐, or kinetics‐based models, but a complementary approach for identifying systems in which composition‐sensitive interactions materially shape molecular assembly or function.

## 11 Experimental, Computational, and Translational Priorities

A priority is to test the framework in well‐characterized interaction systems in which component abundance, binding properties, molecular assembly, and functional output can be measured independently. Experiments should systematically vary the abundances of interacting components across a defined concentration space and, where feasible, perturb interaction affinity while simultaneously measuring assembly and phenotype. Single‐cell, spatial, and time‐resolved approaches will be important when whole‐cell or population measurements do not reflect the molecular pools available for interaction [60, 61, 62, 63].

Computational models should match the experimentally established interaction architecture. Pairwise systems can be described using mass‐action binding, competitive systems require explicit alternative complexes, and cooperative or multivalent assemblies require corresponding higher‐order models. Independent perturbation data should then test whether models that include composition‐sensitive interaction terms or measured assembly states outperform models that treat component abundances independently [4, 5, 12, 29, 30, 31, 32, 33, 34, 36, 37, 38].

Translational studies should follow only where altered relative composition is mechanistically linked to a disease‐associated assembly state. Clinical studies can then test whether composition‐based measurements improve upon individual‐protein biomarkers, while therapeutic studies can ask whether restoring the relevant molecular assembly improves phenotype. The objective is therefore not restoration of a numerical ratio itself, but identification and correction of the molecular state through which altered composition affects function [45, 59].

## 12 Concluding Remarks

Relative protein abundance can provide biologically important information, but a protein ratio does not act independently of the molecular interactions that generate function. The same ratio can produce different assembly states at different absolute concentrations or binding affinities, while changes in affinity can redistribute complexes without altering the measured ratio. The protein‐ratio rheostat is therefore best understood as a framework linking relative composition to molecular assembly within defined biochemical and cellular regimes.

This formulation leads to a more discriminating experimental strategy. Rather than measuring protein ratios in isolation, investigators should, when feasible, determine component abundance, binding or assembly properties, complex composition, and downstream functional output. Perturbations that span the joint concentration space of interacting components and independently alter interaction affinity can then determine which molecular states provide mechanistic or predictive information in a particular system.

Different molecular architectures can generate distinct response behaviors: simple pairwise interactions may produce saturating responses, competition can redistribute components according to abundance and affinity, cooperative systems can generate threshold‐like transitions, and multivalent assemblies can exhibit non‐monotonic or reentrant behavior. There is therefore no universal protein‐ratio response curve. The relevant test is whether explicitly modeling composition‐sensitive interactions or measured assembly states improves mechanistic explanation or predictive performance compared with models that treat component abundances independently.

Viewed in this way, protein ratios are neither substitutes for concentration nor merely descriptive measurements. Relative composition is an experimentally testable coordinate of component abundance whose biological significance depends on the interaction regime in which it is measured. Defining when changes in relative composition alter molecular assembly, and when they do not, provides a rigorous basis for interpreting molecular perturbations and identifying regulatory imbalances that may contribute to disease.

## Author Contributions

**Subbaya Subramanian**: conceptualization, writing – original draft, writing – review and editing. **Reena V. Kartha**: writing – review and editing.

## Conflicts of Interest

The authors declare no conflicts of interest.

## Data Availability

Data sharing is not applicable to this article, as no datasets were generated or analyzed during the current study.
